# The neurodevelopmental profile of healthy children with premature anterior fontanel closure

**DOI:** 10.55730/1300-0144.5393

**Published:** 2022-02-27

**Authors:** Meltem DİREK, Khatuna MAKHAROBLIDZE, Burçin GÖNÜLLÜ POLAT, Asena Ayça ÖZDEMİR, Çetin OKUYAZ

**Affiliations:** 1 Department of Pediatric Neurology, Faculty of Medicine, Mersin University, Mersin, Turkey; 2 Department of Medical Education, Faculty of Medicine, Mersin University, Mersin, Turkey

**Keywords:** Premature fontanel closure, Denver II, Bayley III, neurodevelopment

## Abstract

**Background/aim:**

We aimed to assess the neurodevelopmental status of healthy children with premature anterior fontanel closure.

**Materials and methods:**

This retrospective observational study was conducted on 40 (20 M, 20 F) children admitted to Mersin University Pediatric Neurology Outpatient Clinic between 2015–2020 with complaints of premature fontanel closure. Patients with dysmorphic features, microcephaly, craniosynostosis, hypoxic-ischemic sequelae, infections, metabolic disorders, intracranial hemorrhage, epilepsy, endocrine problems, additional congenital anomalies, intrauterine growth retardation (IUGR), prematurity, and postmaturity were excluded. The Denver II and Bayley III tests were applied to all patients and controls.

**Results:**

The Denver II identified retardations in gross motor skills (p = 0.015) and personal-social skills (p = 0.042) and Bayley III in cognitive (p = 0.030) and motor skills (p = 0.007) in the study group. None of the participants in the study group had neurodevelopmental retardation, according to the Bayley III normal standards.

**Conclusion:**

Our results suggest that children with premature fontanel closure may develop motor retardation. These children should, therefore, be closely monitored for neurodevelopmental aspects.

## 1. Introduction

Cerebral development is rapid in the first two years of life when two-thirds of the brain growth is completed. During this time, the child acquires necessary developmental steps such as sitting, walking, understanding and speaking [1].

Anatomically, there are six fontanels in a normal newborn, including one anterior, one posterior, two mastoids, and two sphenoids. The anterior fontanel is usually assessed with the fontanel examination [2]. Its final closure occurs due to the conversion of fibroblasts to osteoblasts after birth [3]. Premature closure of the fontanel may also be associated with many pathological conditions such as microcephaly, craniosynostosis, and congenital hyperthyroidism. Therefore, in the case of premature closure of the anterior fontanel, the child should be thoroughly examined for accompanying dysmorphic findings, to distinguish normal variants from pathologic conditions [4].

Craniosynostosis develops as a result of early ossification of some or all of the cranial sutures. The premature closure of the cranial sutures in craniosynostosis does not allow the brain to grow; therefore, leads to head shape anomalies, developmental problems, and vision and hearing loss [5]. Plagiocephaly is a shape abnormality of the head and is classified as synostotic or nonsynostotic [6]. Positional plagiocephaly, a variant of normal, is defined as the flattening of a specific area on the baby’s head due to exposure to continuous pressure at one point [7]. However, some developmental problems are increasingly reported in babies with plagiocephaly [8,9]. In a previous study we conducted, we found mild retardation in gross motor development in plagiocephaly cases by using the Denver II. The Denver II, being a developmental screening test, was the limitation of the study [10]. In the present study, suitable cases were selected and evaluated with the Denver II and Bayley III tests.

Our study aimed to assess children’s developmental profiles with early anterior fontanel closure via the Denver II and Bayley III tests comparing them with healthy children. To the best of our knowledge, this is the first study to evaluate children with premature anterior closure using the Bayley III test.

## 2. Methods

The responsible family physicians noticed early fontanel closure in all study participants and referred them to our pediatric neurology outpatient clinic for further examination. The family or family physician had no concerns for the child’s development. Forty patients who applied to Mersin University Pediatric Neurology Outpatient Clinic between 2015–2020 with complaints of early fontanel closure and confirming investigations were retrospectively enrolled in the study.

Our previous study was performed between 2011–2019 with sixty-six patients with a mean age of 7.77 ± 6.50 months (min 2, max 41). We found mild retardation in gross motor development with the Denver II. Because the Denver II is a neurodevelopmental screening test, we conducted this new study with Bayley III to confirm our previous results. In this study, we selected forty new patients with a history of early fontanel closure. We conducted the study under the principles of the Declaration of Helsinki, and ethics approval was obtained from the Clinical Research Ethics Committee of our center [reference number: 2020/675, approval date: 30/09/2020].

### 2.1. Patient information and study design

Forty patients who met the inclusion criteria were included in the study and formed the study group. The control group consisted of 40 healthy children followed up in the healthy children’s outpatient clinic with similar age, gender, socioeconomic and sociocultural levels, whose fontanels were not closed before six months and who did not have any chronic diseases or medication use. The study group, then, was divided into two groups according to the timing of fontanel closure: 1–3 months versus 4–6 months. The results of the Bayley III test were compared between these groups. The Bayley III subtests with scores below 70 are considered as developmental retardation, below 79 as risky developments. Although the Bayley III is not considered as an intelligence quotient test (IQ), it reliably identifies infants with developmental retardation when cognitive, language or total motor scores are below 85.

The inclusion criteria were as follows: term birth (38–42 weeks gestation), birth weight appropriate for gestational age, regular head circumference check and standard head shape and circumference at birth, normal antenatal-natal-postnatal history, normal neurological examination, normal laboratory values (blood count, calcium, phosphorus, alkaline phosphatase, vitamin D, parathyroid hormone, thyroid function tests), no craniosynostosis or microcephaly, no congenital anomalies, no dysmorphic findings, no epilepsy, no hypoxic-ischemic sequelae, no intracranial hemorrhage, no other diseases affecting neurodevelopment, and expected neuroimaging results (brain tomography or brain MRI).

Exclusion criteria were defined as the following: prematurity or postmaturity, intrauterine growth retardation, hospitalization in the neonatal intensive care unit, craniosynostosis, microcephaly, macrocephaly, a congenital anomaly, dysmorphic findings, epilepsy, hypoxic-ischemic sequelae, intracranial hemorrhage, metabolic and endocrine problems, abnormal laboratory tests, abnormal neuroimaging results, and other neurodevelopmental disorders.

Fontanel size was recorded by calculating the arithmetic mean of the sum of measurements of the anterior fontanel from front to back and its transverse ends [11]. Head circumference was measured with a nonflexible paper tape. The measurement was made by passing a tape measure anteriorly over the supraorbital protrusions and posteriorly over the occiput protrusion; the measurements were then noted in millimeters. Those with a head circumference below 3p or −2 standard deviation by age and gender in percentile charts were assumed to have microcephaly and were excluded. The patients’ prenatal-natal-postnatal history, head circumference at birth, anterior fontanel width, fontanel closure time, laboratory test results, neuroimaging findings, and genetic analysis reports were obtained from the records. Head circumference growth charts were examined during follow-up visits to the outpatient clinic. A cranial CT examination was requested in all patients presenting to our outpatient clinic with complaints of premature closure of the fontanel to rule out craniosynostosis. After inclusion in the study group, cerebral MRI, chromosomal analysis, and microarray CGH were requested in all patients with parental consent.

A certified child development professional administered the Denver II and Bayley III tests to children when they were full and had adequate sleep.

### 2.2. Denver Developmental Screening Test II (Denver II)

The Denver II test was performed in all patients. This test compares the skills of the children with their peers in personal-social (PS), fine motor (FMS), language, and gross motor skills (GMS). The validity and reliability of the test were performed by Anlar et al. in Turkey [12]. All subdevelopmental areas and the overall scores are scored. The number of items depends on the age and ability of the child. According to the number of caution and delayed items, the score is designated as normal, suspected, or abnormal. Each subtest score of the patient is evaluated separately. In each subtest, firstly, the number of items the child should achieve with 90% success was determined according to the child’s age. Later, the percentile score was obtained by a formula mentioned below (subtest score = 100 × the number of items/the number of items to succeed to the 90%).

### 2.3. Bayley Scales of Infant Development III (Bayley III)

In children aged 0 to 42 months, the Bayley III is used to assess development in cognitive, language, and motor skills. It is considered as the gold standard test in determining developmental delays in children. The Bayley test was first used in 1969, revised in 1993, and most recently standardized by Bayley in 2006 [13]. The Bayley III measures cognitive development with age-appropriate activities such as counting numbers, completing puzzles, matching colors, and many types of play. The motor scale has two sections: fine motor (FM) and gross motor (GM). Fine motor skills such as visual tracking, reaching out and grasping, and gross motor movements such as sitting, crawling, standing, jumping, climbing stairs, and walking are assessed. The language domain has two parts: receptive language (RL) and expressive language (EL). The language domain assesses spelling, use of body language, describing things, use of vocabulary, plural suffixes, and verb conjugations. In our study, the cognitive, receptive language, total language, fine motor, gross motor, and total motor (TM) developmental scores of the Bayley III test were recorded. Test scores are scaled on a range of 40–160 to give a mean of 100 and an SD of 15 (69 and below: extremely low, 70–79: borderline, 80–89: low average, 90–109: average, 110–119: high average, 120–129: superior, 130 and above: very superior). A Bayley III subtest score below 85 means −1 SDS; below 70 means −2 SDS. In this study, test scores below 70 are accepted as neurodevelopmental retardation, and below 79 as risky neurodevelopment.

### 2.3. Statistical analysis

Statistical analysis of the obtained data was performed using the Statistica 13.5 package program. The propensity score method was used to compare patient and control groups in terms of age and gender. For the effect size of 0.80 (large effect) between the patient and control groups, it was considered appropriate to include at least 26 subjects in each group with 80% power and 5% type I error. Normality control of continuous variables was done with the Shapiro-Wilk test. Since age, maternal age, and the Bayley III conform to the normal distribution, student’s t-test was used to compare means by the two groups. Since the Denver II does not conform to the normal distribution, the Mann-Whitney U test was applied to compare the medians between the two groups. Spearman Rho coefficient was performed in patients with premature fontanelle closure to investigate the relationship between Bayley III and Denver II. Depending on the group, chi-square test was conducted to examine the distribution of gender, mother’s education, father’s education, and head circumference. The Linear-by- Linear Association test was done to compare the Bayley III subtest scores between groups. The statistical significance level was set at p ≤ 0.05.

## 3. Results

The mean Bayley III test age of the study group (20 F, 20 M) and the control group (20 F, 20 M) was 16.92 ± 9.39 months (min: 4.28–max: 42.12) and 16.83 ± 9.39 months (min: 6.24–max: 41.12), respectively. No significant difference was found between the groups in age, gender distribution, maternal age, parental education level, and socioeconomic status (p > 0.05) (Table 1).

Our study group’s mean fontanel closure time was 3.33 ± 1.66 (min 1–max 6) months. Of the 40 patients in the study group, 6 had anterior fontanel closure in the first month, eight in the 2nd month, nine in the 3rd month, and 17 between 4–6 months. None of the participants in the control group had an anterior fontanel closure time of less than six months. The sociodemographic data of the study and control groups and the head circumference percentiles for the Denver II and Bayley III test at admission are shown in Table 1.

The prenatal and postnatal courses, including birth weight, birth length, birth head circumference, laboratory values, neurological examination, head circumference growth chart, and brain CT results, were normal in all patients included in the study. The genetic and brain MRI results of all children were within normal limits. None of the children had chronic diseases that could affect neurodevelopment.

The Denver II total scores were 316.16 ± 6.03 (339–360) in the study group with early fontanel closure and 359.10 ± 1.90 (354–360) in the control group. A significant difference in the Denver II total scores existed between the study and control groups (p = 0.015 by Mann-Whitney U test) (Table 2). Looking at the Denver II subtest results, it was found that the study group’s personal-social and gross motor test scores with premature fontanel closure were significantly lower than those of the control group. Table 2 presents test results and p values.

The Bayley III cognitive and motor subtest scores in the study group were significantly lower than the control group. The test results and p values are presented in Table 3. Table 4 shows distribution of the Bayley III subtest results and p values. There are only two patients who showed risky neurodevelopment in the motor subtest of the Bayley III. No patients showed neurodevelopmental retardation according to Bayley III normal standards.

The Denver II and Bayley III test results did not differ significantly between patients with a fontanel closure time of 1–3 months and 4–6 months (p > 0.005).

## 4. Discussion

In the present study, 40 children (20 M, 20 F) with premature fontanel closure, considered a normal variant, were examined using the Denver II and Bayley III tests. The Denver II identified lower scores in gross motor (p = 0.015) and personal social (p = 0.042) areas and the Bayley III in cognitive (p = 0.030) and motor (p = 0.007) subtests in children with premature fontanel closure than in the control group. Our study is the first study in the literature evaluating these children with Bayley III to the best of our knowledge.

The Bayley and Denver tests are the most commonly used early developmental screening tools. There are many studies in which child developmental stages have been evaluated using the Denver II [14,15]. Thanks to its psychometric properties and quantitative scoring system, the Bayley III test is the instrument of choice to regularly assess neurocognitive development and monitor intervention processes [9].

Neurocognitive and developmental outcomes of craniosynostosis have long been recognized [5]. Synostotic or nonsynostotic plagiocephaly with single suture closure was, for a time, considered a cosmetic problem, with insignificant neurocognitive and developmental consequences [16]. However, Collett et al., Kordestani et al., Panchal et al. reported that plagiocephaly cases, synostotic and nonsynostotic, might show deviations from normal neurocognitive development at school-age [8,17,18]. In light of these findings of plagiocephaly, we searched for possible developmental outcomes in children with premature fontanel closure.

Many studies have reported that factors such as genetics, race, and gender influence the closure time of the anterior fontanel [19,20]. In our study, the anterior fontanel closed at 1 to 3 months in 23 of our 40 patients, and at 4 to 6 months in 17; the mean was 3.33 ± 1.66 (min 1–max 6) months. Pindirik et al. studied the closure time of the anterior fontanel in healthy children with head CT and reported that AF closed in 3%–5% of children at 5 to 6 months [19]. Boran et al. found that the anterior fontanel closed in 11% of infants at three months and 32% at six months [20]. They noted that if head circumference, head shape, and growth rate are normal in infants with premature fontanel closure, normal neurodevelopment is expected [19–21].

In our study group compared to the controls, retardation was found in gross motor (p = 0.015) and PSS (p = 0.042) with the Denver II, and in cognitive (p = 0.030) and motor areas (p = 0.007) with Bayley III (Table 2,3). Our study with the Denver II is the only study in the literature evaluating healthy children with premature fontanel closure from a neurodevelopmental perspective [10].

On the other hand, some studies on children with plagiocephaly may be a source for us. Kordestani et al. showed that in 110 children with plagiocephaly, both mental and psychomotor index scores were lower with the Bayley II test than in the controls [17]. Panchal et al. examined cases with single-suture craniosynostosis and plagiocephaly without synostosis using the Bayley II test [18]. They showed cognitive and psychomotor retardation in synostotic and nonsynostotic cases and concluded that synostotic cases tend to have more pronounced retardation. Collett et al. studied children with plagiocephaly using the Bayley III and Wechsler Individual Achievement Test III (WIAT-3) at different time points and showed retardation of motor functions in early life and academic and cognitive functions at school-age in the patient group compared to the control group [8]. The same study also reported that early identified motor retardation improved with age, but language and cognitive test scores were impaired at older ages. It has been suggested that the impairment in language and cognitive skills may be related to the inability of children to adequately explore their environment due to motor retardation at an early age.

It is not known why the anterior fontanel closes prematurely in some children. Craniosynostosis is thought to be related to problems in genes affecting FGFR and downstream signaling (MAPK, JAK-STAT) pathways. MAPK stimulates cell growth and division, and JAK-STAT stimulates the differentiation of fibroblasts into osteoblasts [22]. It is unknown why gross motor and cognitive skills are impaired in healthy children with synostotic or nonsynostotic plagiocephaly. Premature fontanel closure and plagiocephaly could affect gyri growth. In addition, genetic variation in the FGFR, MAPK, and JAK-STAT pathways may be associated with premature fontanel closure and neurodevelopmental retardation.

In the present study, retardation in motor, language, and cognitive skills was assessed using the Bayley III. Although none of the participants in the study group had neurodevelopmental retardation (Table 4), a statistically significant distribution difference was found in the Bayley III motor subtests (Table 4). On the other hand, the mean scores of the motor and cognitive subtests were significantly lower in the study group (Table 3). The neurocognitive developmental status showed no significant difference between children with fontanel closure at 1 to 3 months and at 4 to 6 months (p > 0.05). This result suggests that the timing of fontanel closure is not the primary determinant of neurocognitive developmental outcome. A neurocognitive developmental difference may be related to genetic differences rather than the inhibition of gyri growth. In our previous study with the Denver II, retardation was found only in GM skills. In the present study, retardation was assessed in GM and PS skills with the Denver II and motor and cognitive skills with the Bayley III. The difference between the results of our first study and this study is probably related to the mean age of the study cases. The mean age of cases in our former study was 7.77 ± 6.50 (2–41) months, whereas the mean age was 16.02 ± 9.39 (4.28–42.12) months in the current study. The difference in the results of our two studies is consistent with the observational study of children with plagiocephaly by Collet et al. in which motor skills were impaired at an early age, and language and cognitive skills were impaired at an older age [8]. In contrast to Collet et al., we did not find any retardation of language skills in our study. This could be due to the younger age of our study group than Collet et al.’s study group.

The strengths of our study are: (1) we found a motor developmental delay in children with premature fontanel closure, which is considered a normal variant, (2) this is the first study to evaluate this group of patients with the Bayley III test, (3) we used a control group in our study, although the tests have normative data, and (4) we also confirmed that the neurocognitive developmental differences were found in cases with nonsynostotic plagiocephaly. The limitations of our study are the retrospective selection and observational evaluation of cases, single-center setting, and the lack of more accurate assessment tools for cognitive functioning with intelligence and neuropsychological tests.

In conclusion, our study results indicate that motor, personal-social, and cognitive retardations may develop in children with premature fontanel closure, which is considered a normal variant. Therefore, the need to follow these children for neurocognitive and developmental aspects should be kept in mind. Our results need to be verified by broader community-based prospective studies in which children are monitored with detailed neuropsychological test batteries.

## Figures and Tables

**Table 1:** Clinical characteristics of patients with premature AF closure **(**study group) and control group.

	Study group (n: 40)	Control group (n: 40)	p
Gender (Female/male)	20/20	20/20	1.00
Age mean ± SD, months (min-max)	16.92 ± 9.39 (4.28–42.12)	16.90 ± 9.27 (6.24–41.12)	0.992
Mother’s educational level None	3(7.5)	4(10)	0.916
Primary	18(45)	20(50)	
Secondary	11(27.5)	9(22.5)	
Higher/high	8(20)	7(17.5)	
Mother’s age, years (mean)	30.95 ± 6.13	30.97 ± 5.09	0.984
Father’s educational level None	4(10)	5(12.5)	0.608
Primary	12(30)	17(42.5)	
Secondary	12(30)	9(22.5)	
Higher/high	12(30)	9(22.5)	
Father’s age, years (mean)	32.6 ± 6.78	31.8 ± 5.43	0.562
Head circumference a)3–10p	13(32.5)	4(10)	0.153
b)10–25p	12(30)	13(32.5)	
c)25–50p	8(20)	13(32.5)	
d)50–75p	5(12.5)	8(20)	
e) > 5p	2(5)	2(5)	
AF closure time, months (mean) a) at the 1st month	6(15)	None	-
a) at the 2nd month	8(20)	None	
b) at the 3rd month	9(22.5)	None	
c) at 4 to 6 months	17(42.5)	None	

**Table 2:** Comparison of Denver II results of patients with premature fontanel closure (study group) with the control group.

	Study group		Control group		
Denver II	Mean ± SD (min-max)	Median [%25–%75]	Mean ± SD (min-max)	Median [%25–%75]	p
PSS	89.38 ± 2.06 (80–90)	90 [90–90]	90.00 ± 0.00 (90–90)	90 [90–90]	0.042
FMS	89.95 ± 0.32 (88–90)	90 [90–90]	90.00 ± 0.00 (90–90)	90 [90–90]	0.317
Lang.	89.29 ± 2.13 (79–90)	90 [90–90]	89.70 ± 1.32 (84–90)	90 [90–90]	0.155
GMS	87.45 ± 4.49 (69–90)	90 [86–90]	89.40 ± 1.50 (84–90)	90 [90–90]	0.015
Total Denver score	316.16 ± 6.03 (339–360)	360 [353–360]	359.10 ± 1.90 (354–360)	360 [360–360]	0.015

AF: anterior fontanels, DDST II: Denver Developmental Screening Test II, FMS: fine motor score, GMS: gross motor score, PSS: personal social score p: Mann Whitney U test.

**Table 3:** Comparison of the Bayley III results of patients with premature fontanel closure (study group) with the control group.

Bayley III	Study group Mean ± SD (min-max)	Control group Mean ± SD (min-max)	p
Cog. score	99.13 ± 7.42 (85–115)	103.18 ± 8.94 (90–130)	0.030
Lang. score	103.05 ± 9.51 (89–124)	106.43 ± 10.91 (91–138)	0.144
Motor score	99.6 ± 10.45 (76–118)	105.35 ± 8.13 (91–133)	0.007

Cog. score: cognitive score, Rec. Lang.: receptive language, Exp. Lang.: expressive language, Lang. Score: language score, p: student’s t-test

**Table 4:** The distribution of Bayley III subtest scores of the study and the control group.

		Study group		Control group		Total		
		n	%	n	%	n	%	p
Cognitive score	Very superior	0	0.0	2	5.0	2	2.5	0.069
	Superior	0	0.0	0	0	0	0	
	High average	6	15.0	8	20.0	14	17.5	
	Average	32	80.0	30	75.0	62	77.5	
	Low average	2	5.0	0	0.0	2	2.5	
	Borderline	0	0.0	0	0.0	0	0	
	Extremely low	0	0.0	0	0.0	0	0	
Language score	Very superior	0	0.0	2	5.0	2	2.5	0.157
	Superior	2	5.0	1	2.5	3	3.8	
	High average	7	17.5	10	25.0	17	21.3	
	Average	29	72.5	27	67.5	56	70.0	
	Low average	2	5.0	0	0.0	2	2.5	
	Borderline	0	0.0	0	0.0	0	0	
	Extremely low	0	0.0	0	0.0	0	0	
Motor score	Very superior	0	0.0	1	2.5	1	1.3	0.005
	Superior	0	0.0	0	0.0	0	0.0	
	High average	9	22.5	16	40.0	25	31.3	
	Average	25	62.5	23	57.5	48	60.0	
	Low average	4	10.0*	0	0.0	4	5.0	
	Borderline	2	5.0	0	0.0	2	2.5	
	Extremely low	0	0.0	0	0.0	0	0	

p: Linear-by-Linear Association

* means the higher rate

## Abbreviations

AF: Anterior fontanel
FGFR: Fibroblast growth factor receptors
JAK-STAT: Janus activated protein kinase-signal transducers and activators of transcription
MAPK: Mitogen-activated protein kinase

## References

[1] Özer S Kazancı NÖ Karaaslan E Yılmaz R Fontanel değerlendirmesi (Evaluation of Fontanelles) Pediatric Practice and Research 2013 1 4 9 (in Turkish with an abstract in English)

[2] Carlo WA Physical Examination of Newborn Infant Kliegman RM Nelson Textbook of Pediatrics 19th ed USA 2011

[3] Kiesler J Ricer R The abnormal fontanel American Family Physician 2003 67 2547 2552 12825844

[4] Tunnessen WW Roberts KB Signs and Symptoms in Pediatrics 3nd ed Philadelphia Lippincott Williams & Wilkins 1999

[5] Kajdic N Spazzapan P Velnar T Craniosynostosis-recognition, clinical characteristics, and treatment Bosnian Journal of Basic Medical Science 2018 18 110 116 10.17305/bjbms.2017.2083 PMC5988529 28623672

[6] Martiniuk A Vujovich-Dunn C Park M Yu W Lucas BR Plagiocephaly and developmental delay: a systematic review Journal of Developmental Behavioral Pediatrics 2017 38 67 78 10.1097/DBP.0000000000000376 28009719

[7] Bock FD Braunn V Renz-Polster H Deformational plagiocephaly in normal infants: a systematic review of causes and hypotheses Archives Disease in Childhood 2017 102 535 542 10.1136/archdischild2016-312018 28104626

[8] Collett BR Wallace ER Kartin D Cunningham ML Speltz ML Cognitive Outcomes And Positional Plagiocephaly Pediatrics 2019 143 e20182373 10.1542/peds.2018-2373 30635350 PMC6361360

[9] Collett BR Wallace ER Kartin D Matthew LS Infant/toddler motor skills as predictors of cognition and language in children with and without positional skull deformation Child’s Nervous System 2019 35 157 163 10.1007/s00381-018-3986-4 PMC6447299 30377774

[10] Sarıgeçili E Makharoblidze K Direk Çobanoğulları M Yıldırım DD Kömür M Okuyaz Ç Neurodevelopmental risk evaluation of premature closure of the anterior fontanelle Child’s Nervous System 2021 37 561 566 10.1007/s00381-020-04846-6 32737565

[11] Popich GA Smith DW Fontanels: range of normal size The Journal of Pediatrics 1972 80 749 752 10.1016/s0022-3476(72)80125-2 5018385

[12] Eratay E Bayoğlu B Anlar B Preschool developmental screening with denver II test in semi-urban areas J Pediatr Child Care 2015 1 2 4

[13] Bayley N Scales of Infant and Toddler Development 3rd ed San Antonio, TX The Psychological Corporation 2006

[14] Durmazlar N Öztürk Ç Ural B Karaağaoğlu E Anlar B Turkish children’s performance on denver II: effect of sex and mother’s education Developmental Medicine & Child Neurology 2008 40 411 416 10.1111/j.1469-8749.1998.tb08217.x 9652783

[15] Worku BN Abessa TG Wondafrash M Vanvuchelen M Bruckers L Kolsteren P Granitzer M The relationship of undernutrition/psychosocial factors and developmental outcomes of children in extreme poverty in Ethiopia BMC Pediatrics 2018 18 45 10.1186/s12887-018-1009-y 29426302 PMC5809114

[16] Martiniuk A Jacob J Faruqui N Yu W Positional plagiocephaly reduces parental adherence to SIDS guidelines and inundates the health system Child: Care, Health and Development 2016 42 941 950 10.1111/cch.12386 27504717

[17] Kordestani RK Patel S Bard DE Gurwitch R Panchal J Neurodevelopmental delays in children with deformational plagiocephaly Plastic and Reconstructive Surgery 2006 117 207 218 10.1097/01.prs.0000185604.15606.e5 16404269

[18] Panchal J Amirsheybani H Gurwitch R Cook V Francel P Neas B Levine N Neurodevelopment in children with single suture craniosynostosis and plagiocephaly without synostosis Plastic and Reconstructive Surgery 2001 108 1492 1498 10.1007/s00381-006-0251-z 11711916

[19] Pindrik J Ye X Ji BG Pendleton C Ahn ES Anterior fontanelle closure and size in full-term children based on head computed tomography Clinical Pediatrics (Phila) 2014 53 1149 1157 10.1177/0009922814538492 24920348

[20] Perran Boran P Oğuz F Furman A Sakarya S Evaluation of fontanel size variation and closure time in children followed up from birth to 24 months Journal of Neurosurgery Pediatrics 2018 22 323 329 10.3171/2018.3.PEDS17675 29856300

[21] Pedroso FS Rotta N Quintal A Giordani G Evolution of anterior fontanel size in normal infants in the first year of life Journal of Child Neurology 2008 23 1419 1423 10.1177/0883073808319318 19073847

[22] Terwisscha van Scheltinga AF Bakker SC Kahn RS Kas MJH Fibroblast growth factors in neurodevelopment and psychopathology Neuroscientist 2013 19 479 494 10.1177/1073858412472399 23343917

